# Berry syndrome, a rare congenital cardiac structural abnormality with 1-stage surgical repair: A case report

**DOI:** 10.1097/MD.0000000000041009

**Published:** 2025-01-03

**Authors:** Yueqiu Su, Zhou Leng

**Affiliations:** a Department of Anesthesiology, West China Hospital, Sichuan University/West China School of Nursing, Chengdu, Sichuan, China; b Department of Anesthesiology, West China Hospital, Sichuan University, Chengdu, Sichuan, China.

**Keywords:** aortopulmonary window, Berry syndrome, interruption of the aortic arch

## Abstract

**Rationale:**

Berry syndrome is a complex congenital heart anomaly characterized by a combination of aortopulmonary window, interrupted aortic arch or hypoplastic aortic arch or coarctation of the aorta, anomalous origin of the right pulmonary artery, patent ductus arteriosus, and intact ventricular septum. It is an extremely rare condition, with approximately 100 reported cases to date.

**Patient concerns:**

In this article, we report a case of a 6-year-old girl with Berry syndrome who presented with significant ischemic and hypoxic symptoms.

**Diagnoses:**

Based on the patient’s medical history and examinations, a definitive diagnosis of Berry syndrome was established.

**Interventions:**

She underwent a 1-stage surgical treatment and had a successful discharge. The surgical process was aimed to preserve the growth potential of the aorta and pulmonary arteries while achieving unobstructed left ventricular outflow and continuity of the aortic arch, ensuring no shunting between the main pulmonary arteries.

**Outcomes:**

Intraoperative transesophageal echocardiography confirmed continuous flow through the aortic arch and no shunting between the MPA and the aorta.

## 1. Introduction

Berry syndrome is a rare syndrome characterized by a combination of congenital heart structural abnormalities, including aortopulmonary window (APW), aortic origin of the right pulmonary artery, patent ductus arteriosus (PDA), interrupted aortic arch (IAA) or hypoplastic aortic arch or coarctation of the aorta, and intact ventricular septum. Since its initial description by Berry et al in 1982,^[1]^ only about 100 cases have been reported in the English literature to date.^[2]^

Patients with Berry syndrome often present with severe pulmonary hypertension, and the right pulmonary artery dilates due to continuous left-to-right shunting, leading to potential airway compression symptoms.^[3]^ On the other hand, lower limb perfusion in these patients relies on the ductus arteriosus and collateral circulation, making them prone to lower limb ischemic symptoms.^[4]^ Most patients are diagnosed prenatally or in infancy,^[5]^ and may present with cyanosis, respiratory distress, and prominent cardiac murmurs. The majority of patients require immediate surgical intervention at birth. Here, we report a case of a 6-year-old girl with Berry syndrome. She was diagnosed with congenital heart disease at birth but was not confirmed to have Berry syndrome until the age of 6 when she underwent surgical treatment.

## 2. Case report

**Figure 1.**
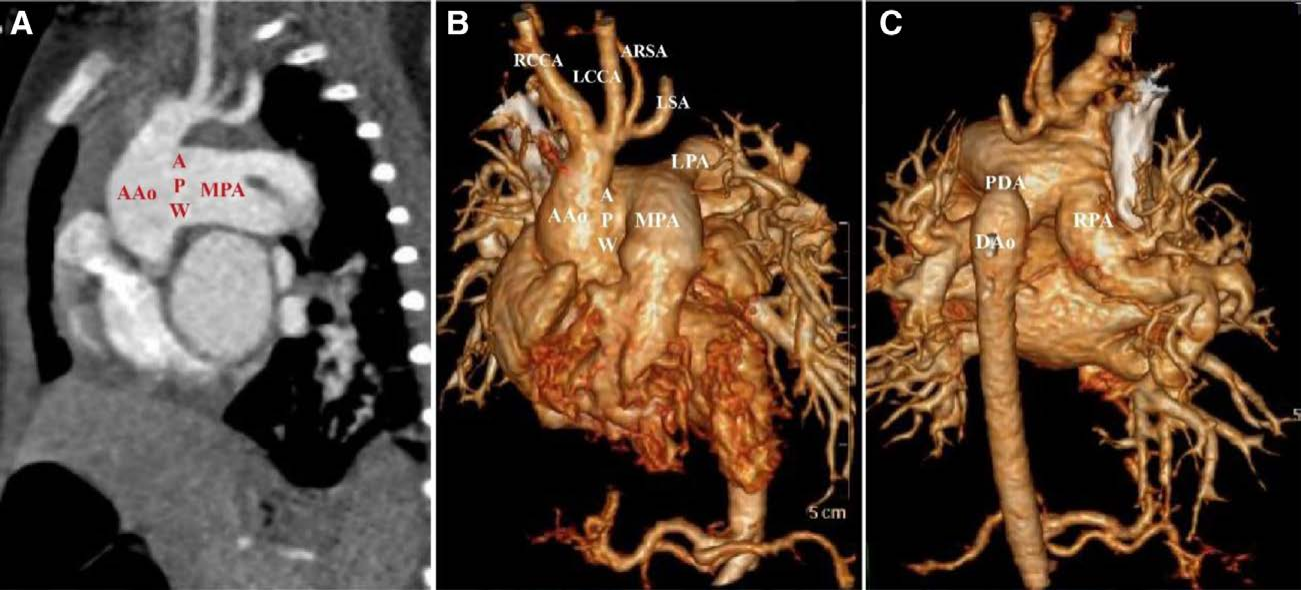
(A) CTA revealed a large aortopulmonary window between ascending aorta and main pulmonary. (B) Three-dimensional reconstruction demonstrated type A interrupted aortic arch and 4 branches of aortic arch. (C) Three-dimensional reconstruction revealed aortic origin of the right pulmonary artery and a large patent ductus arteriosus. CTA = computed tomography angiography.

The patient is a 6-year-old girl from a farming family in a non-high-altitude area. She was presented with mild cyanosis of the lips at birth and was initially diagnosed with congenital heart disease. Because of progressive cyanosis and dyspnea, the patient came to our outpatient clinic for further diagnosis and treatment. She had normal growth and development without significant physical or intellectual delays. The family reported that she was prone to pneumonia and frequently coughed since birth. Physical examination revealed significant cardiac murmurs, clubbing of the fingers, and cyanosis of the lips. Electrocardiography showed sinus rhythm with biventricular hypertrophy and right axis deviation. Transthoracic echocardiography revealed significant hypertrophy of both ventricles and the left atrium, with an absent echo between the left wall of the ascending aorta and the right wall of the main pulmonary artery (MPA), which measured approximately 18 mm. There was a narrow segment in the isthmus of the aortic arch, measured approximately 4mm, and interruption of the aortic arch distal to the left subclavian artery (LSA). The right pulmonary artery (RPA) arose from the ascending aorta, while the left pulmonary artery (LPA) originated from the pulmonary trunk. Between the descending aorta and the left pulmonary artery, there was a prominent PDA with a diameter of approximately 6 mm. The atrial and ventricular septa were intact, valve function was normal, and the coronary artery origins were normal. The pulmonary hypertension was 50 mm Hg. The patient did not take pulmonary antihypertensive treatment before surgery. Subsequently, the patient underwent CT angiography, which confirmed the findings of the echocardiography. The 3-dimensional reconstruction clearly showed a type I APW, type A IAA, and PDA. Four branch vessels were also visualized, namely the right common carotid artery (RCCA), left common carotid artery (LCCA), anomalous right subclavian artery (ARSA), and LSA, which all arose from the proximal end of the interrupted aortic arch (Fig. 1A–C).

Based on the patient’s medical history and examinations, a definitive diagnosis of Berry syndrome was established, and surgical intervention was indicated. The patient’s family requested surgery. A standard mid-sternotomy approach was performed, revealing abundant collateral vessels from the main pulmonary artery. During the repair of interrupted aortic arch, conventional deep hypothermic circulatory arrest and selective cerebral perfusion were employed. Firstly, the ductus arteriosus was dissected distally and ligated, followed by excision of the ductal tissue. The aortic arch and descending aorta were carefully mobilized, and an end-to-side anastomosis was performed between the descending aorta and the aortic arch. The anterior wall was reinforced using a pericardial patch. After the correction of IAA, the RPA was disconnected. The APW was observed, and the defect was approximately 1cm from the aortic valve annulus. The origin of the coronary arteries was normal. Along the edge of the APW, aorta and MPA were separated, while the RPA was end-to-side anastomosed to the MPA. A bovine pericardial patch was used, and the incision in the aorta was closed with 6-0 prolene sutures in a continuous manner. Intraoperative transesophageal echocardiography confirmed continuous flow through the aortic arch and no shunting between the MPA and the aorta. The patient received further treatment in the ICU postoperatively and was transferred to a regular ward after 5 days. The pulmonary hypertension reduced to 26 mm Hg after surgery and no pulmonary hypertension medication was needed. The patient did not need hemodynamic support after the surgery. A successful evaluation was conducted, and the patient was discharged 7 days later.

## 3. Discussion

Berry syndrome is an extremely rare congenital condition, accounting for only 0.046% of reported cases of congenital heart structural abnormalities.^[6]^ Patients are often diagnosed prenatally or in infancy, and may present with cyanosis, respiratory distress, respiratory tract infections, cardiac murmurs, heart failure, and other systemic symptoms.^[2]^ Hu et al^[5]^ suggested that patients should undergo immediate surgical intervention upon detection, even in the neonatal period, in order to avoid irreversible pulmonary hypertension and progressive right heart failure, which could have detrimental effects. Additionally, early surgical correction could restore normal blood supply to the lower body.

The currently widely accepted theory regarding the formation of Berry syndrome suggests that a significant shunt between the aorta and pulmonary artery leads to reduced blood flow in the aortic isthmus during fetal development.^[7]^ Failure of the aortic septum results in an aortopulmonary septal defect, which can be located at the proximal or distal end of the septum. Failure of posterior truncal septation may disrupt normal blood flow and cause the pulmonary bifurcation to attach incorrectly to the unseptated truncus arteriosus instead of connecting to the main pulmonary artery. As a result, the RPA becomes connected to the aorta, while the LPA becomes connected to the pulmonary trunk.^[8]^ According to research by Bi et al,^[2]^ the majority of Berry syndrome patients have type II APW and type A IAA.

Echocardiography is a commonly used method for diagnosing complex congenital heart diseases. As mentioned earlier, some patients with Berry syndrome can be diagnosed prenatally through echocardiography. However, these patients are often missed because they usually have a normal 4-chamber heart and outflow tract appearance. The use of the 3-vessel view (3VV) can improve the prenatal detection rate of Berry syndrome. Additionally, APW is typically easily detected by echocardiography, therefore, Zhang et al^[6]^ suggested that when APW is detected, it is necessary to determine the origin of the RPA and the morphology of the aortic arch. For postnatal infants, as these patients often exhibit easily noticeable symptoms, TTE has become the primary tool for evaluating Berry syndrome.^[4]^ Computed tomography angiography (CTA) with 3-dimensional reconstruction and cardiac magnetic resonance imaging (MRI) can provide comprehensive anatomical information in patients with Berry syndrome.^[9,10]^

Surgical treatment of Berry syndrome primarily focuses on repairing the anatomical abnormalities of APW and IAA. Currently, multiple surgical approaches have been discussed in various centers.^[3,5,11]^ In general, the surgical process should aim to preserve the growth potential of the aorta and pulmonary arteries while achieving unobstructed left ventricular outflow and continuity of the aortic arch, ensuring no shunting between the main pulmonary arteries.

## 4. Conclusion

In this case, we performed an end-to-side anastomosis between the aortic arch and descending aorta and directly reimplanted the right pulmonary artery onto the main pulmonary artery, ensuring normal blood flow and preserving the growth potential of the pulmonary artery. Three months later, the patient’s first follow-up examination showed favorable results.

## Author contributions

**Conceptualization:** Yueqiu Su, Zhou Leng.

**Data curation:** Yueqiu Su, Zhou Leng.

**Formal analysis:** Yueqiu Su, Zhou Leng.

**Investigation:** Yueqiu Su, Zhou Leng.

**Methodology:** Yueqiu Su, Zhou Leng.

**Project administration:** Yueqiu Su, Zhou Leng.

**Resources:** Yueqiu Su, Zhou Leng.

**Software:** Yueqiu Su, Zhou Leng.

**Supervision:** Yueqiu Su, Zhou Leng.

**Validation:** Yueqiu Su, Zhou Leng.

**Visualization:** Yueqiu Su, Zhou Leng.

**Writing – original draft:** Yueqiu Su, Zhou Leng.

**Writing – review & editing:** Yueqiu Su, Zhou Leng.
